# Predictive factors for percutaneous nephrolithotomy bleeding risks

**DOI:** 10.1016/j.ajur.2022.02.003

**Published:** 2022-02-20

**Authors:** U Phun Loo, Chun Hou Yong, Guan Chou Teh

**Affiliations:** Urology Department, Sarawak General Hospital, Kuching, Malaysia

**Keywords:** Percutaneous nephrolithotomy, Predictive factor, Risk factor, Bleeding, Blood loss

## Abstract

**Objective:**

This study aimed to identify predictive factors for percutaneous nephrolithotomy (PCNL) bleeding risks. With better risk stratification, bleeding in high-risk patient can be anticipated and facilitates early identification.

**Methods:**

A prospective observational study of PCNL performed at our institution was done. All adults with radio-opaque renal stones planned for PCNL were included except those with coagulopathy, planned for additional procedures. Factors including gender, co-morbidities, body mass index, stone burden, puncture site, tract dilatation size, operative position, surgeon's seniority, and operative duration were studied using stepwise multivariate regression analysis to identify the predictive factors associated with higher estimated hemoglobin (Hb) deficiency.

**Results:**

Overall, 4.86% patients (*n*=7) received packed cells transfusion. The mean estimated Hb deficiency was 1.3 (range 0–6.5) g/dL and the median was 1.0 g/dL. Stepwise multivariate regression analysis revealed that absence of hypertension (*p*=0.024), puncture site (*p*=0.027), and operative duration (*p*=0.023) were significantly associated with higher estimated Hb deficiency. However, the effect sizes are rather small with partial eta-squared of 0.037, 0.066, and 0.038, respectively. Observed power obtained was 0.621, 0.722, and 0.625, respectively. Other factors studied did not correlate with Hb difference.

**Conclusion:**

Hypertension, puncture site, and operative duration have significant impact on estimated Hb deficiency during PCNL. However, the effect size is rather small despite adequate study power obtained. Nonetheless, operative position (supine or prone), puncture number, or tract dilatation size did not correlate with Hb difference. The mainstay of reducing bleeding in PCNL is still meticulous operative technique. Our study findings also suggest that PCNL can be safely done by urology trainees under supervision in suitably selected patient, without increasing risk of bleeding.

## 1 Introduction

Percutaneous nephrolithotomy (PCNL) has mostly replaced open surgery for renal stone management. PCNL is the present gold standard for symptomatic patients with renal stone burden of more than 20 mm [1]. Evolutions and innovations in surgical skills, instruments, and technologies over the past decades have led to ever-increasing range of stone sizes applicable for its use. It is now considered reasonable option for smaller-than-traditional PCNL stone sizes. Despite these improvements, bleeding remains one of the most concerned post-operative complication. Most of the time, post-PCNL bleeding can be managed conservatively with or without blood transfusion. A small proportion of patients require further intervention such as angiographic embolization.

The aim of this study was to identify predictive factors for PCNL bleeding risks. With better risk stratification, bleeding in high-risk patient can be anticipated and facilitates early identification which translates to reduction in post-operative morbidities and mortality.

## 2 Patients and methods

This study is registered with National Medical Research Registry (NMRR ID: NMRR-19-4145-51656) and has been approved by Medical Research Ethics Committee Malaysia. All adults aged 18 years old and above with radio-opaque stones are included in this study excluding those with coagulopathy or planned additional procedures such as ureteroscopy lithotripsy, bilateral PCNL, and vesicolithotripsy. All patients were admitted 2 days prior to operation. Pre-operative laboratory tests including complete blood count were taken. Stone burden was assessed pre-operatively by translating the shape of renal stones on a radiograph onto a grid graph paper to obtain the surface area of stones. The demographics, investigation results, and procedural data were prospectively collected.

All patients received prophylactic antibiotics according to local guidelines of the hospital. All PCNLs were performed under general anesthesia, either in supine or prone position based on surgeon's preference. In patients planned for supine PCNL, they were retained in Galdakao-modified Valdivia position throughout the operation. Whereas for PCNL in prone position, they were first positioned in lithotomy for retrograde catheterization with 6 Fr open ended ureteric catheter under fluoroscopy guidance using a rigid cystoscope. Subsequently patients were re-positioned, and all other parts of the procedures were completed in prone position. Percutaneous renal access was obtained with the aid of C-arm. Tracts were dilated using Amplatz serial renal dilator set. Stone disintegration was achieved using ultrasound lithotriptor and/or pneumatic lithoclast followed by retrieval of stones using forceps and/or basket. Additional tracts were created when indicated at the same session. At the end of the procedure, a 12 Fr or 14 Fr nephrostomy tube was placed in most of the cases.

Post-operative complete blood count was obtained on post-operative Day 1. Hemoglobin (Hb) difference was obtained by subtracting post-operative Hb from pre-operative Hb. Each packed cell transfused intra-operatively is factored to the estimated blood loss with an approximation that 1 unit of blood transfused increases Hb level by 1.0 g/dL. Factors contributing to the bleeding risk during PCNL were identified by comparison of age, gender, ethnicity, obesity, hypertension, diabetes, chronic kidney disease, pre-operative anemia, stone size, staghorn disease, puncture number, puncture site, tract dilatation size, operative position, surgeon's seniority, and operative duration.

An obese person is defined as one with body mass index equal or more than 30 kg/m^2^ according to WHO [2]. Anemia is defined by the WHO as a Hb level below 12 g/dL in women and 13 g/dL in men [3]. Stone types were categorized as staghorn calculi for stones with any branch occupying more than one calyx of the collecting system and non-staghorn calculi [4]. Chronic kidney disease is defined as kidney damage for 3 months or more, irrespective of cause. It can be staged from 1 to 5 according to glomerular filtration rate (mL/min/1.73 m^2^), 90 or greater, 60 to 89, 30 to 59, 15 to 29, and less than 15, respectively [5].

The minimum number of sample size is 114 based on the study by Tabachnick and Fidell [6] who suggested a guideline for sample size for multiple linear regression 50 + 8 × 8 (number of predictive factors).

The association of estimated blood loss with various factors studied was evaluated with stepwise multivariate regression analysis using SPSS version 23 (IBM Corp., Armonk, New York, USA). The *p*-value of <0.05 was considered significant.

## 3 Results

A total of 144 patients who underwent elective PCNL in Sarawak General Hospital were recruited from January 2019 to January 2020. Median age of patients was 56 (range 21–82) years. Median stone size was 1155 (range 116–8300) mm^2^. Staghorn stone was detected in 29.9% (*n*=43) of the patients. A total of 117 (81.2%) patients were treated with single-access PCNLs, and the remaining 27 (18.8%) patients underwent multi-access procedures. The median operative duration was 100 (range 30–385) min. Detailed demographics and clinical data are summarized in Table 1, Table 2.

**Table 1:** Patient characteristics affecting post-PCNL estimated Hb difference by univariate analysis (*n*=144).

Patient demographic and characteristic	Value^a^	Hb, mean difference, g/dL	*p*-Value
Age, year	56 (21–82)	NA	0.181
Gender			0.79
Female	70 (48.6)	1.279	
Male	74 (51.4)	1.228	
Ethnicity			0.301
Malay	35 (24.3)	1.054	
Ethic Chinese	23 (16.0)	1.387	
Iban	63 (43.8)	1.297	
Bidayuh	13 (9.0)	1.765	
Others	10 (6.9)	0.723	
Obesity			0.414
Yes	48 (33.3)	1.144	
No	96 (66.7)	1.307	
Hypertension			0.027
Yes	92 (63.9)	1.096	
No	52 (36.1)	1.531	
Diabetes			0.279
Yes	48 (33.3)	1.108	
No	96 (66.7)	1.325	
CKD			0.505
Stage 1	20 (13.9)	0.885	
Stage 2	54 (37.5)	1.300	
Stage 3	57 (39.6)	1.330	
Stage 4	9 (6.2)	1.478	
Stage 5	4 (2.8)	0.850	
Pre-operative anemia			0.120
Yes	39 (27.1)	1.013	
No	105 (72.9)	1.342	

PCNL, percutaneous nephrolithotomy; Hb, hemoglobin; CKD, chronic kidney disease; NA, not applicable.

a Values are presented as median (range) or *n* (%).

**Table 2:** Clinical factors affecting post-PCNL estimated Hb difference by univariate analysis (*n*=144).

Clinical characteristic	Value^a^	Hb, mean difference, g/dL	*p*-Value
Stone size, mm^2^	1155 (116–8300)	NA	0.171
Staghorn disease			0.207
Yes	43 (29.9)	1.435	
No	101 (70.1)	1.175	
Puncture number			0.010
1	117 (81.2)	1.137	
≥2	27 (18.8)	1.756	
Puncture site			0.008
Lower pole	65 (45.1)	1.223	
Mid pole	43 (29.9)	0.984	
Upper pole	33 (22.9)	1.500	
Renal pelvis	3 (2.1)	3.033	
Tract size, Fr			0.085
16–20	25 (17.4)	0.900	
21–30	119 (82.6)	1.327	
Operative position			0.571
Prone	84 (58.3)	1.300	
Supine	60 (41.7)	1.187	
Surgeon's seniority			0.095
Trainee under supervision	45 (31.2)	1.489	
Consultant	99 (68.8)	1.146	
Operative duration, min	100 (30–385)	NA	0.001

PCNL, percutaneous nephrolithotomy; Hb, hemoglobin; NA, not applicable.

a Values are presented as median (range) or *n* (%).

**Table 3:** Factors affecting post-PCNL estimated Hb difference: outcomes of multivariate logistic regression analysis.

Factors	*p*-Value	Partial eta-squared	Observed power
Hypertension	0.024	0.037	0.621
Puncture number	0.523	0.003	0.098
Puncture site	0.027	0.066	0.722
Operative duration	0.023	0.038	0.625

PCNL, percutaneous nephrolithotomy; Hb, hemoglobin.

Overall, 4.86% (*n*=7) patients received packed cells transfusion. The mean estimated Hb deficiency was 1.3 (range 0–6.5) g/dL and the median was 1.0 g/dL. One patient post-surgical outcome was complicated with renal artery pseudoaneurysm requiring angioembolization. No incidence of bowel injury, pneumothorax, or mortality was captured during this period of study.

Stepwise multivariate regression analysis revealed that absence of hypertension (*p*=0.024), puncture site (*p*=0.027), and operative duration (*p*=0.023) were significantly associated with higher estimated Hb deficiency. However, the effect sizes are rather small with partial eta-squared of 0.037, 0.066, and 0.038, respectively. Observed power obtained was 0.621, 0.722, and 0.625, respectively (Table 3). Presence of hypertension appears to be protective for bleeding risk, with a mean estimated Hb deficiency of 1.096 g/dL among patients with hypertension as compared to 1.531 g/dL among those without. Puncture to renal pelvis is associated with higher Hb difference followed by puncture to upper pole, lower pole, and middle pole with a mean Hb difference of 3.033, 1.500, 1.223, and 0.984 g/dL, respectively. Also, longer operative time is associated with greater estimated Hb deficiency.

Among all the patient-related factors, age, gender, ethnicity, obesity, diabetes, chronic kidney disease, pre-operative anemia, stone surface area, or staghorn stone did not correlate with Hb difference. Of the procedural-related factors, surgeon's seniority, operative position, puncture number, or tract dilatation size did not correlate with Hb difference.

## 4 Discussion

PCNL is the treatment choice for most renal stones, conventionally for stones larger than 20 mm; nevertheless, it is now applicable for smaller stones ever since the introduction of miniaturized PCNL. However, this procedure is associated with several complications including bleeding necessitating blood transfusion. The transfusion rate is 4.86% within our cohort. This finding is comparable to the transfusion rates reported in the literature available which ranged from 3% to 23% [[7], [8], [9], [10], [11], [12], [13]]. Often the decision of blood transfusion intra-operatively are made by anesthesiology team. This result could be attributable to the advancement in surgical instruments, improved surgical techniques, and the possible differences in transfusion strategies.

Estimations of stone burden using two-dimensional surface area are relatively crude as compared to more sophisticated software driven estimates. Nonetheless, in our study, stone size and presence of staghorn did not have significant correlation with estimated Hb deficiency. PCNL for large and complex stones are usually performed by consultant urologist instead of trainee, which is a possible confounding factor. On top of that, stone configuration or complexity was not captured within this study. For example, small infundibular stone and large simple renal stone could have similar bleeding risk despite differences in stone burden.

The prevalence of diabetes mellitus and hypertension in Malaysia are 18% and 35.3%, respectively [14,15]. In a study by Akman et al. [16] in 2011, these comorbidities were found to be associated with higher blood transfusion rate in PCNL. However, more recent studies showed no relationship between bleeding risk and diabetes or hypertension [8,17]. Among the patient-related factors, hypertension was observed to associate with lower estimated Hb deficiency in our study. We found no correlation among different stages of chronic kidney disease, diabetes, or estimated Hb deficiency. Contrary to the popular belief, we found higher body mass index did not have impact on estimated Hb deficiency in patients undergoing PCNL.

Among the procedure-related factors, puncture site (*p*=0.027) and operative duration (*p*=0.023) are associated with larger Hb differences based on the outcome of multivariate logistic regression analysis. Only 2.1% of patients had access via renal pelvis and this is associated with the highest estimated Hb deficiency, followed by puncture to upper pole, lower pole, and middle pole with mean estimated Hb deficiencies (g/dL) of 3.033, 1.500, 1.223, and 0.984, respectively. Direct puncture into renal pelvis substantially increases the bleeding risk and should be avoided at all times. Access via upper calyceal may traumatize the posterior segmental artery during puncture and is associated with higher bleeding risk as compared to middle and lower calyceal puncture [16]. However, upper calyceal puncture has advantages to achieve good access into majority of calices and renal pelvis, even upper ureter at times.

Multiple access tracts are often required to tackle complex and large renal stone inside complex caliceal system. Multiple punctures for multiple access tracts cause renal tissue trauma and increase the risk of renal vascular injury thus increasing the risks of bleeding. Three studies reported number of access tracts as a significant risk for post-operative bleeding [8,18,19], whereas other two found this relationship not statistically significant [9,20]. Also, miniaturization of the PCNL has continued to gain popularity in the past decade. It is now an option for those with smaller-than-traditional PCNL stone sizes. Ruhayel et al. [21] reported significantly reduced blood loss in the expense of longer operative time in miniaturized PCNL. In our study, we performed 81.2% of the PCNL using single tract access and multiple tract procedures in 18.8% of the patients. Standard PCNL with access tracts dilatation ranging from 26 Fr to 30 Fr were observed in 82.6% patients whereas 17.4% had mini-PCNL where access tracts were dilated up to 18 Fr. The number of access used and tract dilatation size did not have significant impact on the estimated Hb deficiency. All of our access tracts were dilated serially using Amplatz dilatation system which uses beveled-edge fascial dilator to spread the parenchymal tissue rather than lacerating it.

There are increasing studies that are suggesting long hours of operation is associated with higher incidence of complication [22]. This was also found true for PCNL based on PCNL Global Study database by Yamaguchi et al. [23] from Clinical Research Office of the Endourological Society. It shows that the operating time does have impact on bleeding complication. Likewise, we observed the same relationship where longer operative time is associated with greater estimated Hb deficiency.

Falahatkar and his team [24] reported a lower incidence of blood transfusion in the supine group with comparable stone-free rate between supine and prone position. Whereas in a systematic review by Birowo and colleagues [25], they found that there is no significant difference in the rate of transfusion between these two groups. Similarly, we observed that operative position, whether supine or prone has no significant impact on estimated Hb deficiency (*p*=0.571). It remains debatable whether supine or prone PCNL has better outcomes and the decision for each approach should be based on surgeon experience and patient characteristics.

At our center, PCNLs are performed by consultant urologists and also by trainee urologists under supervision. Difficult operations such as PCNL for large and complex stones are reserved for consultant urologists, while trainee urologists will attempt for simple renal stones. The mean estimated Hb deficiency in PCNLs performed by trainees is 1.489 g/dL and those done by consultant urologist is 1.146 g/dL. However, this difference is not statistically significant (*p*=0.095). This finding suggests with appropriate case selection, trainee may safely perform PCNL under proper supervision and guidance. Allen and colleagues [26] proposed 60 PCNL procedures for surgical competence and 115 procedures for excellence.

We recognize there are several limitations within our study. Not all patients had CT scan done prior to operation. Thus, our measurements of stone burden were based on surface area of stone obtained from radiograph. However, this is the best representation of stone burden within our resource limitation. Also, stone composition is not studied at our center due to limited resources and it is usually reserved to be done in recurrent stone formers. Lastly, decisions for blood transfusion were often made by anesthesiology team, and the threshold was not reported.

## 5 Conclusion

Hypertension, puncture site, and operative duration have significant impact on estimated Hb deficiency during PCNL. However, the effect size is rather small despite adequate study power obtained. Nonetheless, operative position (supine or prone), puncture number, or tract dilatation size did not correlate with Hb difference. The mainstay of reducing bleeding in PCNL is still meticulous operative technique. Our study findings also suggest that PCNL can be safely done by urologist trainees under supervision in suitably selected patient, without increasing risk of bleeding.

## Author contributions

*Study design*: U Phun Loo, Chun Hou Yong, Guan Chou Teh.

*Data acquisition*: U Phun Loo, Chun Hou Yong.

*Data analysis*: U Phun Loo, Chun Hou Yong, Guan Chou Teh.

*Drafting of manuscript*: U Phun Loo.

*Revision of the manuscript*: U Phun Loo, Chun Hou Yong, Guan Chou Teh.

## Conflicts of interest

The authors declare no conflict of interest.
